# Fabrication of enzyme-based coatings on intact multi-walled carbon nanotubes as highly effective electrodes in biofuel cells

**DOI:** 10.1038/srep40202

**Published:** 2017-01-05

**Authors:** Byoung Chan Kim, Inseon Lee, Seok-Joon Kwon, Youngho Wee, Ki Young Kwon, Chulmin Jeon, Hyo Jin An, Hee-Tae Jung, Su Ha, Jonathan S. Dordick, Jungbae Kim

**Affiliations:** 1 Center for Environment, Health and Welfare Research, Korea Institute of Science and Technology, Seoul 02792, Republic of Korea; 2 Department of Energy and Environmental Engineering, Korea University of Science and Technology (UST), Seoul 02792, Republic of Korea; 3 Department of Chemical and Biological Engineering, Korea University, Seoul 02841, Republic of Korea; 4 Department of Chemical and Biological Engineering, Rensselaer Polytechnic Institute, Troy, NY 12180, USA; 5 Department of Chemical and Biomolecular Engineering, Korea Advanced Institute of Science and Technology, Daejeon 34141, Republic of Korea; 6 The Gene and Linda Voiland School of Chemical Engineering and Bioengineering, Washington State University, Pullman, WA 99164-2710, USA; 7 Green School, Korea University, Seoul 02841, Republic of Korea

## Abstract

CNTs need to be dispersed in aqueous solution for their successful use, and most methods to disperse CNTs rely on tedious and time-consuming acid-based oxidation. Here, we report the simple dispersion of intact multi-walled carbon nanotubes (CNTs) by adding them directly into an aqueous solution of glucose oxidase (GOx), resulting in simultaneous CNT dispersion and facile enzyme immobilization through sequential enzyme adsorption, precipitation, and crosslinking (EAPC). The EAPC achieved high enzyme loading and stability because of crosslinked enzyme coatings on intact CNTs, while obviating the chemical pretreatment that can seriously damage the electron conductivity of CNTs. EAPC-driven GOx activity was 4.5- and 11-times higher than those of covalently-attached GOx (CA) on acid-treated CNTs and simply-adsorbed GOx (ADS) on intact CNTs, respectively. EAPC showed no decrease of GOx activity for 270 days. EAPC was employed to prepare the enzyme anodes for biofuel cells, and the EAPC anode produced 7.5-times higher power output than the CA anode. Even with a higher amount of bound non-conductive enzymes, the EAPC anode showed 1.7-fold higher electron transfer rate than the CA anode. The EAPC on intact CNTs can improve enzyme loading and stability with key routes of improved electron transfer in various biosensing and bioelectronics devices.

Carbon nanotubes (CNTs) have gathered great attention due to their unique physical, chemical and electrical properties, which allows for their use in nanoelectronics^1^,^2^,^3^, nanocomposites^4^,^5^, nanolithography^6^,^7^, biosensing^8^,^9^,^10^,^11^, drug delivery^12^,^13^,^14^,^15^, and cancer targeting treatment^15^,^16^,^17^. However, aggregation and poor dispersion of hydrophobic CNTs in hydrophilic aqueous solution makes their versatile uses difficult, especially in aqueous-based bio-related applications, by limiting effective interaction of biomolecules with CNTs. Various techniques have been proposed to improve the dispersion of CNTs in aqueous buffer solution^18^,^19^,^20^. For example, the dispersion of CNTs was improved via covalent or non-covalent functionalization^21^,^22^, chemical oxidation using strong acids^23^,^24^, plasma treatment^25^, polymer wrapping^26^,^27^, surfactant addition^28^,^29^,^30^, and DNA or protein addition^31^,^32^,^33^,^34^,^35^,^36^,^37^,^38^. In bio-related applications, CNTs are usually treated with strong acids, generating hydrophilic carboxyl groups on the surface of CNTs, which allows for dispersion of CNTs in aqueous solution and can be used to provide carboxylic acid groups for the chemical attachment of biomolecules. However, acid treatment of CNTs is not only tedious and time-consuming, but also causes structural defects that can seriously impair electrical conductivity of CNTs^19^,^39^.

In the present work, we report the simple dispersion of CNTs without acid treatment by adding CNTs directly into an enzyme solution, and their use for facile enzyme immobilization. We hypothesize that good dispersion of CNTs can be attributed to the amphiphilic nature of an enzyme’s surface^40^,^41^, where hydrophobic moieties enable interaction with the hydrophobic CNT surface while hydrophilic moieties interact with the aqueous solution, thereby preventing CNT aggregation and leading to effective CNT dispersion. Based on this phenomenon of CNT dispersion in an enzyme-containing solution, we have developed a novel protocol of enzyme immobilization and stabilization, called “enzyme adsorption, precipitation, and crosslinking (EAPC)”. The first step of enzyme adsorption represents dispersion of CNTs in the enzyme solution, which is followed by the sequential steps of enzyme precipitation and chemical crosslinking. We prepared EAPCs of glucose oxidase (GOx) on intact CNTs and investigated the resulting conjugate morphology, activity and stability. Immobilized and stabilized GOx in the form of EAPC was also employed to fabricate an enzyme anode for biofuel cells. Even though enzymatic biofuel cells have a great potential as a small power source for implantable devices as well as biosensors, their practical applications are being hampered by their short lifetime and low power output^42^,^43^,^44^. The successful incorporation of EAPC with high enzyme loading and stability can lead to the development of highly-effective enzyme electrodes by improving both lifetime and power density of biofuel cells.

## Results and Discussion

### Preparation of EAPC on CNTs

**Figure 1:**
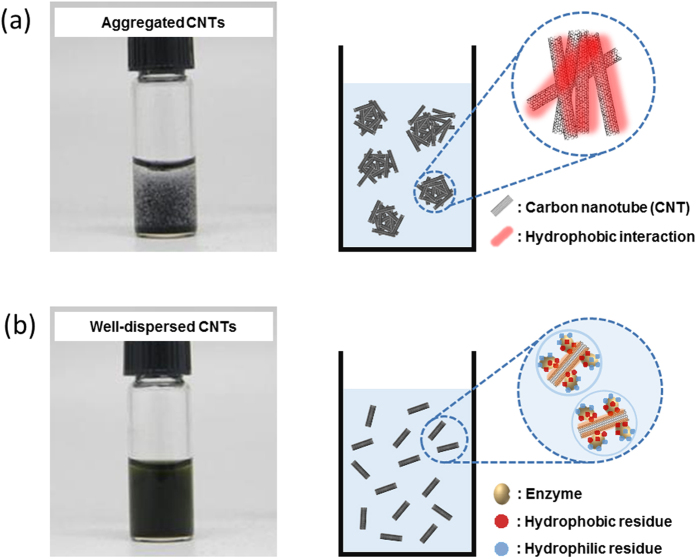
Comparison of the CNT dispersion in the absence and presence of enzymes. (**a**) Aggregated CNTs in aqueous solution, and (**b**) well-dispersed CNTs in enzyme solution (10 mg/mL GOx), together with hypothetical schematics for both CNT aggregation in aqueous solution and dispersed CNTs in enzyme solution.

Figure 1 shows the aggregation of native multi-walled carbon nanotubes (CNTs) in 100 mM phosphate buffer (PB, pH 7.0) and the good apparent dispersion of CNTs in GOx solution (10 mg/ml GOx in 100 mM PB, pH 7.0). While hydrophobic native CNTs undergo significant aggregation in PB, good CNT dispersion is observed in the presence of GOx (Fig. 1b). It may be hypothesized that the surfactant-like, amphiphilic nature of the GOx surface facilitates this CNT dispersion. According to 3D structural analysis, GOx has a hydrophobic patch on its surface that can interact with the hydrophobic side wall of intact CNTs, resulting in facile GOx adsorption onto the surface of CNTs (Fig. S1). Concomitantly, hydrophilic interactions between water molecules and hydrophilic side chains on the surface of GOx lead to highly-dispersed intact CNTs in aqueous enzyme solution (Figs S1 and 1b). To test the hypothesis of CNT-GOx and GOx-water interactions enabling both good enzyme adsorption onto CNTs and dispersability in aqueous solution, we added an equal volume of hexane to aqueous GOx solutions containing well-dispersed intact CNTs at various GOx concentrations (0 to 10 mg/ml). Interestingly, only in the absence of GOx, were all of the intact CNTs extracted into the hexane phase. On the other hand, even with the lowest GOx concentration (0.1 mg/ml), intact CNTs were not extracted into the hexane phase, and the CNTs were dispersed in aqueous GOx solution (Fig. S2). When considering the hydrophobic nature of the intact CNT surface, the lack of CNT extraction into the hexane phase in the presence of GOx strongly supports our hypothesis of hydrophobic interaction between CNT surface and hydrophobic patch on the GOx surface that can allow for the retention of CNTs in the enzyme solutions. We performed experiments to determine the dispersion of intact CNTs in aqueous solutions of trypsin (TR), chymotrypsin (CT), horseradish peroxidase (HRP), and bovine serum albumin (BSA) (Fig. S3a). Intact CNTs were well dispersed in each of the protein solutions while CNTs aggregated without protein added to the solution. We also assessed the effect of protein solutions on dispersing various graphene particles. As shown in Fig. S3, similar results were obtained as that for CNTs. These results strongly support our hypothesis of CNT dispersion in aqueous protein solution based on CNT-protein and protein-water interactions, and this could be extended to graphene particles (Fig. S3b).

**Figure 2:**
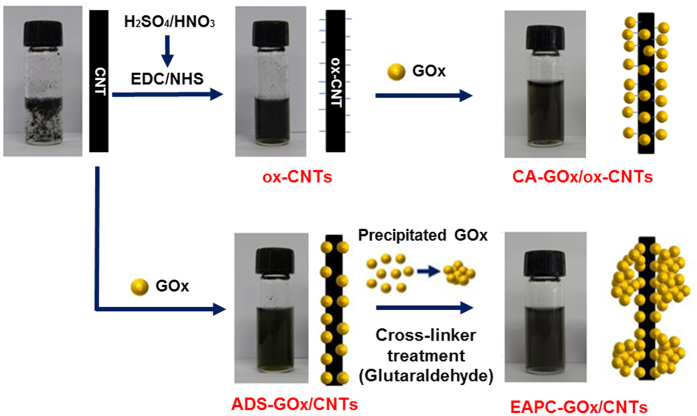
Schematic diagrams for the preparation of CA-GOx/ox-CNTs (CA) and EAPC-GOx/CNTs (EAPC).

To take advantage of well-dispersed and intact CNTs in an aqueous enzyme solution for the development of a unique enzyme immobilization method, we first performed two additional steps of enzyme precipitation and crosslinking after dispersing intact CNTs in aqueous GOx solution. To that end, we proceeded to precipitate GOx by adding ammonium sulfate, and this step was followed by enzyme crosslinking with glutaraldehyde (Fig. 2a). For comparison, we also prepared covalently-attached GOx (CA) on acid-treated CNTs (ox-CNTs) by employing the EDC/NHS linker chemistry to conjugate the carboxyl groups on ox-CNTs with the amino groups on GOx (Fig. 2b).

**Figure 3:**
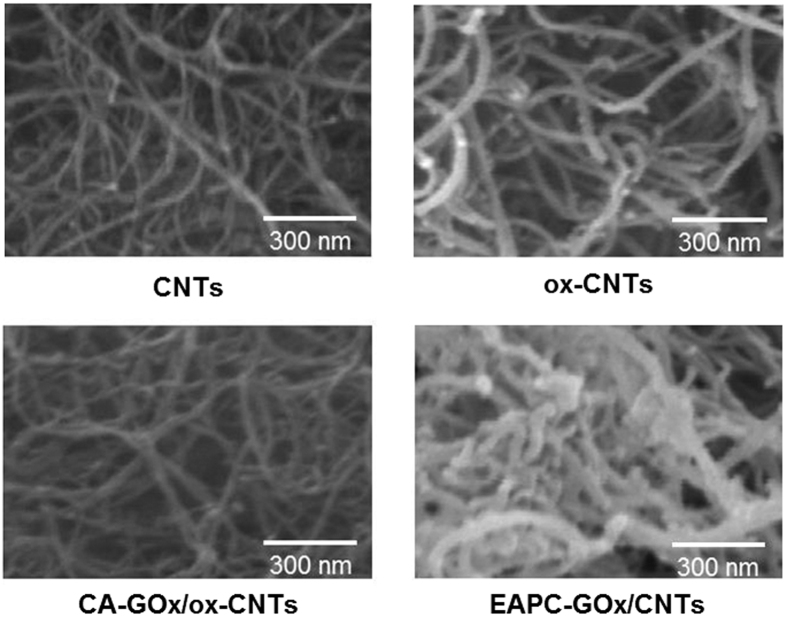
SEM images of CNTs, ox-CNTs, CA-GOx/ox-CNTs, and EAPC-GOx/CNTs. The white bar in each image represents 300 nm.

Scanning electron microscopy (SEM) images of EAPC revealed a thick coating of crosslinked GOx aggregates on the surface of CNTs, which were not observed in the SEM images of other control samples such as CNTs, ox-CNTs and CA (Fig. 3). According to analyses of 20 randomly-selected tubular nanoparticles in the SEM images (Fig. 3), the average thickness of tubular nanoparticles in the samples of CNTs, ox-CNTs, CA and EAPC were 30 ± 10, 35 ± 5, 36 ± 7 and 52 ± 13 nm, respectively (Table S1). Since the thickness of CNTs is estimated to be 30 ± 10 nm, the thickness of crosslinked GOx coatings over CNTs in the samples of EAPC can be estimated to be 22 nm. Because the size of GOx (6.0 × 5.2 × 3.7 nm) can be estimated to be ~5.0 nm^45^, the estimated EAPC thickness of 22 nm represents 4~5 layers of crosslinked GOx aggregates.

### Activity and stability of EAPC-GOx on CNTs

**Figure 4:**
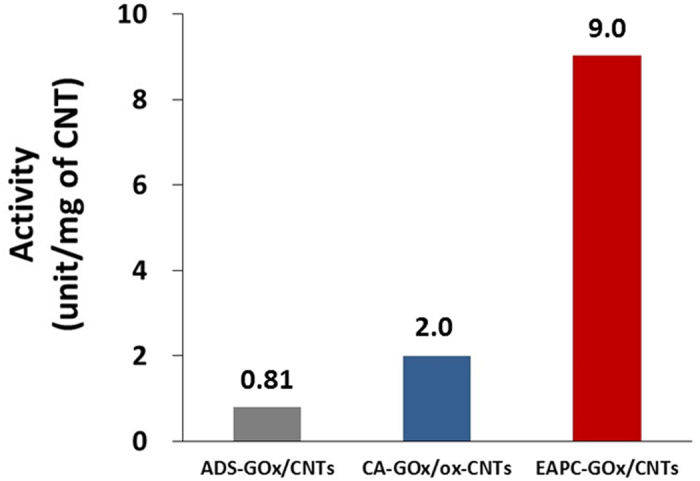
Activities of ADS-GOx/CNTs, CA-GOx/ox-CNTs, and EAPC-GOx/CNTs in an aqueous buffer solution (100 mM sodium phosphate, pH 7.0). GOx activity was measured by the time-dependent increase of absorbance at 500 nm, which represents the oxidation of *o*-dianisidine catalyzed by peroxidase using hydrogen peroxide generated by GOx-catalyzed glucose oxidation.

The activity of immobilized GOx was measured by the oxidation of *o*-dianisidine, which is catalyzed by peroxidase using the hydrogen peroxide generated from GOx-catalyzed glucose oxidation. The measured activities of ADS, CA and EAPC were 0.81, 2.0 and 9.0 units per mg of CNTs (Fig. 4). The activity of EAPC was approximately 12- and 4.5-times higher than those of ADS and CA, respectively. This improved activity of EAPC may be explained by enhanced enzyme loading when compared to ADS and CA, as observed in the SEM images (Fig. 3). Interestingly, the activity ratio of 4.5 matches well with the estimated 4~5 layers of crosslinked GOx aggregates from the SEM images of EAPC and CNTs. We also prepared enzyme-CNT conjugates without the precipitation step, e.g., enzyme adsorption and crosslinking (EAC). The activity of EAC was 2.3 units per mg of CNTs, which is 3.9-fold lower activity than that of EAPC. Hence, enzyme precipitation is important to achieve high enzyme loadings in the form of EAPC.

**Figure 5:**
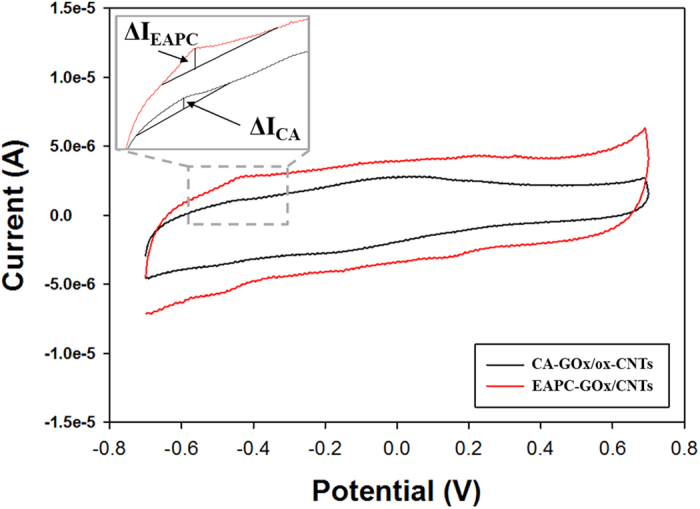
Cyclic voltammograms (CVs) of CA-GOx/ox-CNTs and EAPC-GOx/CNTs with 200 mM glucose in aqueous buffer solution (100 mM sodium phosphate, pH 7.0). The scan rate was 50 mV s^−1^.

Cyclic voltammograms (CVs) are typically used to measure the electrochemical activity of various types of nanobiocatalytic materials. As shown Fig. 5, the peak currents of CA from its baseline (△I_CA_) and EAPC from its baseline (△I_EAPC_) were 0.48 and 0.85 μA, respectively. The peak current of EAPC was 1.8-fold higher than that of CA. This enhanced electrochemical activity of EAPC can be explained by its intact nature of CNTs where its intrinsically high electron conductivity is maintained during its synthesis process. On the other hand, the oxidized CNTs used in CA sample lead to its lower electrochemical activity due to their damaged surfaces and decreased electron conductivity.

**Figure 6:**
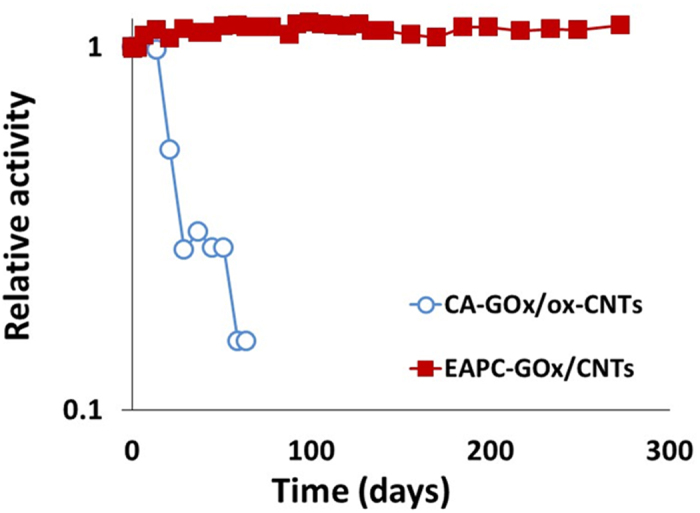
The stabilities of ADS-GOx/CNTs, CA-GOx/ox-CNTs and EAPC-GOx/CNTs in aqueous buffer solution (100 mM sodium phosphate, pH 7.0) at room temperature. Relative activity is defined by the ratio of residual activity at each time point to the initial activity of each sample.

Figure 6 shows the stabilities of ADS, CA and EAPC in an aqueous solution at room temperature. The relative activity is defined as the ratio of residual activity at each time point to the initial activity of each sample. ADS and CA showed a monotonous decrease of GOx activity, while EAPC exhibited no activity loss for 270 days. The inactivation of ADS can be explained by the denaturation of GOx, likely because the interaction between GOx and CNT is not sufficiently strong to prevent enzyme denaturation over extended use. CA has covalent linkages between GOx and CNT, which can prevent the detachment of GOx from the CNT. However, based on the method used, the number of chemical linkages on each GOx molecule is expected to be small, and the prevention of enzyme denaturation cannot be anticipated. On the other hand, the improved stability of EAPC suggests that the multi-point covalent linkages of enzyme molecules effectively prevent enzyme molecules within EAPCs from being structurally denatured and leached away from the EAPC matrix.

### Biofuel cell application of EAPC-GOx on CNTs

**Figure 7:**
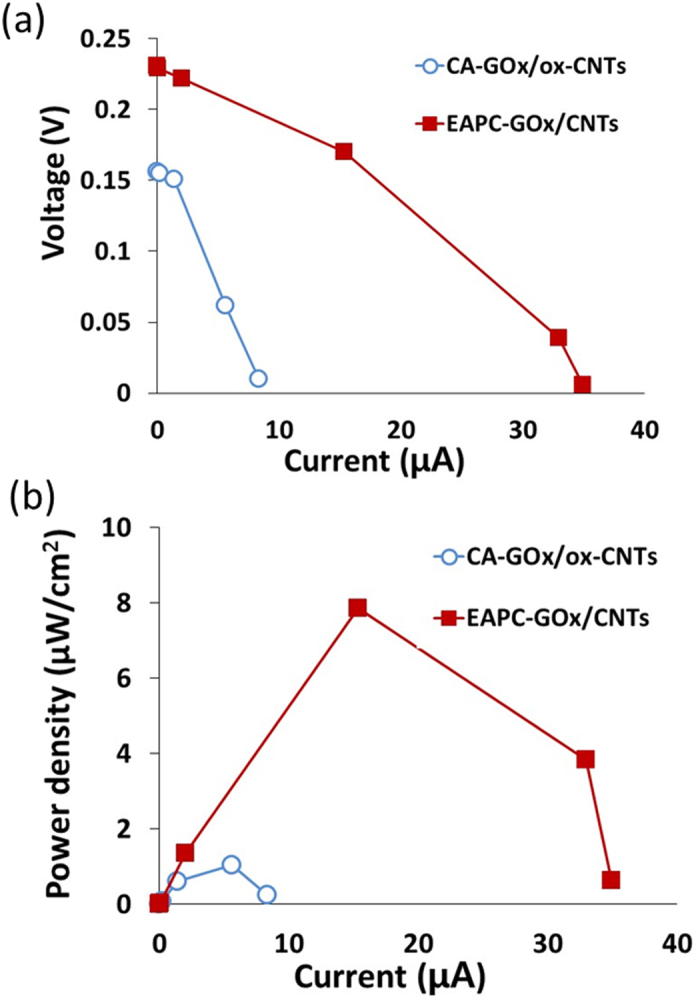
The voltage–current (**a**) and power density-current (**b**) curves of biofuel cells using the enzyme anodes of CA-GOx/ox-CNTs and EAPC-GOx/CNTs.

To exploit the potential applications of highly stable EAPC-GOx/CNTs with high enzyme loading and activity, we prepared an enzyme anode using EAPC that can be integrated into a prototype biofuel cell^46^. GOx has potential uses in electrochemical applications, including biosensors^10^,^47^,^48^ and biofuel cells^44^,^49^,^50^,^51^,^52^,^53^,^54^,^55^,^56^. In addition, recent studies indicate that a good interface between GOx and CNTs is a critical factor in the efficient transfer of electrons from the GOx active site flavin to CNTs^10^,^57^,^58^. For comparison purposes, a CA-based enzyme anode was also prepared. To fabricate these enzyme-containing anodes, CA and EAPC were applied onto carbon papers (CPs) via Nafion entrapment to integrate into the fuel cell module. The electrochemical performance of each anode was measured in terms of current-voltage characteristic (V-I) plots (Fig. 7a). According to the V-I plots, the biofuel cell with the EAPC anode produced a higher open cell potential (OCP), a less ohmic overpotential (i.e., a lower slope of its V-I plot) and higher maximum current density output compared to that with the CA anode. Power density plots were also constructed based on these V-I plots (Fig. 7b). The power density outputs of the biofuel cell with EAPC anode were higher than that with CA anode for the entire current density range. Furthermore, the biofuel cell with EAPC anode produced 7.5 times higher maximum power density output than that with CA anode.

The electrochemical performance enhancement of the EAPC-based anode can be analyzed in terms of electron generation rate and electron transfer rate. It is reasonable to assume that the electron generation rate in enzyme electrodes is proportional to the enzyme activity per unit weight of CNTs^59^,^60^,^61^. As shown in Fig. 4, the enzyme activity of EAPC per unit weight of CNT is higher than that of CA. Because we fixed the weight loading of CNTs for both CA- and EAPC-based enzyme anodes in our biofuel cell tests, the number of electrons generated per unit time in the biofuel cell with the EAPC anode should be higher than that with the CA anode. However, the electrons generated at the active-site center of GOx during glucose oxidation must be collected at the current collector to produce a net power output. To this end, the electron transfer rate constant can be used to quantify how efficiently each enzyme electrode can transfer the electrons from the enzyme active sites to the backbone electrode of carbon paper. Laviron’s simulation, based on CVs at various scan rates, was employed to obtain electron transfer rate constants of the CA and EAPC anodes (Fig. S4)^62^. The electron transfer rate constants of CA and EAPC were 1.8 and 3.1 s^−1^, respectively (Table 1); hence, the electron transfer rate constant of EAPC anode was 1.7 times higher than that of CA anode. This suggests that the EAPC electrode is more efficient in transferring electrons from the enzyme active sites to the backbone electrode even though it contains a higher number of non-conductive enzyme molecules in the form of crosslinked enzyme clusters. In other words, EAPC-based biofuel cells generate more electrons and transfer these electrons more efficiently to the current collector than CA-based biofuel cells. Consequently, the biofuel cell with EAPC anode showed higher electrochemical performance than that with CA anode.

It is possible that the enhanced electrochemical performance of EAPC-based biofuel cells is largely a result of its higher enzyme loading than CA-based biofuel cells. If so, the open circuit potential (OCP) should be similar for both types of enzyme electrodes. However, the OCP of EAPC-based biofuel cell is 1.5-fold higher than that of its CA-based counterpart, which indicates that the EAPC intrinsic electronic properties (e.g., electron transfer rate constant) are improved. Even though theoretical OCP values are a thermodynamic property, experimental values in working fuel cells are strongly influenced by kinetic parameters, including the charge transfer rate due to the small amount of current leakage. The ohmic overpotential for the EAPC anode is also lower than that of the CA-anode as shown in their V-I plots. Furthermore, the maximum power density ratio between EAPC-based and CA-based biofuel cells (7.2) is higher than their enzyme activity ratio (4.5) (Table 1). This difference can be explained by the difference in their electron transfer rate constants. When the activity ratio between EAPC and CA samples is multiplied by their electron transfer rate constant ratio (1.7), the multiplied value (7.6) is similar to their maximum power density ratio under the biofuel cell operating condition (7.2). This analysis suggests that the higher power output of the EAPC anode vs. the CA anode results from the combined effects of higher electron generation rate and improved electron transfer efficiency offered by the EAPC sample. The lower electron transfer rate constant of CA electrode can be explained by the damage of CNTs upon acid treatment, which lowers the electron conductivity of CNTs^39^. As a result, intact CNTs used in the EAPC anode have higher intrinsic electrical conductivity than ox-CNTs used in the CA anode.

In summary, simple dispersion of intact CNTs in aqueous enzyme solutions represents an effective protocol for the facile enzyme immobilization on intact CNTs. When considering the tedious and time-consuming surface functionalization of CNTs for their use in aqueous solution, this simple protocol of CNT dispersion in the operating enzyme solution can renovate the protocols of enzyme immobilization on CNTs. As an example, in the present work we demonstrated the successful dispersion of CNTs in GOx solutions with simultaneous adsorption of the enzyme onto the CNTs followed by precipitation and crosslinking. Such EAPCs of GOx resulted in highly active and stable form of immobilized enzyme preparations at high enzyme loading. The prevention of CNT defects by obviating the tedious acid-treatment step for increasing the hydrophilicity, and hence dispersibility, of CNTs is likely to be helpful in taking full advantage of CNT electron conductivity in various electrochemical applications. As an example, the EAPC of GOx showed improved electron conductivity in a biofuel cell operation when compared to conventional enzyme immobilization of covalent enzyme attachment using acid-treated and oxidized CNTs. The versatile uses of CNTs are well established and still growing, and it is anticipated that the simple CNT dispersion in enzyme solutions can find additional applications of biomolecules in the development of biosensing and bioelectronics devices.

## Methods

### Materials

Glucose oxidase (GOx) from *Aspergillus niger*, trypsin (TR) from porcine pancreas, chymotrypsin (CT) from from bovine pancreas, horseradish peroxidase (HRP), and bovine serum albumin (BSA), sulfuric acid (H_2_SO_4_), nitric acid (HNO_3_), sodium phosphate monobasic, sodium phosphate dibasic, Tris-HCl, Trizma base, 2-(N-morpholino)ethanesulfonic acid (MES), glutaraldehyde (GA), ammonium sulfate, β-D-glucose, *o*-dianisidine and Nafion^®^ were purchased from Sigma Aldrich (St. Louis, MO, USA). N-hydroxysulfosuccinimide (NHS) was obtained from Alfa Aesar (Ward Hill, MA, USA), while N-ethyl-N’-(3-dimethylaminopropyl) carbodiimide hydrochloride (EDC) was purchased from Pierce (Rockford, IL, USA). CNTs (multi-walled, 30 ± 15 nm in outer diameter and 1~5 μm in length, purity >95%) were purchased from Nanolab Inc. (Newton, MA, USA), while graphenes (3 nm in average flake thickness and 10 μm in average diameter) were purchased from Graphene Laboratories Inc. (Calverton, NY, USA). A carbon paper (CP) and membrane electrode assembly (MEA) were purchased from Fuel Cell Store (San Diego, CA, USA).

### Preparation of ADS, CA, EAC and EAPC on CNTs

CNTs (2 mg) in 2 mL of phosphate buffer (PB, 100 mM, pH 7.0) were mixed with 1 mL of GOx solution (10 mg/mL in 100 mM PB, pH 7.0). The mixture was incubated at room temperature under shaking (200 rpm) for 1 h and incubated at 4 °C overnight. We denote this process as the enzyme adsorption (EA) of GOx (ADS-GOx/CNTs, ADS).

For preparation of covalently-attached GOx on CNTs (CA-GOx/ox-CNTs, CA), CNTs were incubated in the acid solution, consisting of H_2_SO_4_ (98%, 7.5 mL) and HNO_3_ (70%, 2.5 mL), at room temperature under shaking (200 rpm) overnight. Acid-treated CNTs were washed with distilled water, dried at 80 °C in a vacuum oven, and stored at room temperature. Acid-treated and dried CNTs (ox-CNTs, 20 mg) were suspended in distilled water (10 mL), and then added to a mixture of MES buffer (4 mL, 500 mM, pH 6.5), NHS aqueous solution (4 mL, 434 mM), and EDC aqueous solution (2 mL, 53 mM). After rigorous stirring at room temperature for 1 h, the suspension was excessively washed with 100 mM MES buffer (pH 6.5). CA was prepared by adding EDC-NHS conjugated CNTs (2 mg/mL) to the 1 mL of GOx solution (10 mg/mL in 100 mM PB, pH 7.0). The mixture was then incubated at room temperature under shaking (200 rpm) for 1 h and incubated at 4 °C overnight.

For the preparation of enzyme adsorption, precipitation, and crosslinking on CNTs (EAPC-GOx/CNTs, EAPC), ammonium sulfate (22 wt% for GOx) was added to the CNTs (2 mg) pre-dispersed in 1 mL of GOx (10 mg/mL), and the mixture was incubated at room temperature for 30 min. After the enzyme precipitation step, crosslinking was performed by adding GA to the mixture at a final concentration of 0.25% (w/v) GA to generate EAPC-GOx. As a control, enzyme adsorption and crosslinking (EAC) on CNTs (EAC-GOx/CNTs, EAC) was prepared without the enzyme aggregation step by omitting the addition of ammonium sulfate for EAPC. The other conditions for preparation of EAC were the same as those for the preparation of EAPC. After GA addition, the samples were incubated at room temperature under shaking (200 rpm) for 30 min, followed by incubation at 4 °C overnight. After overnight incubation, all GOx immobilized samples were treated with Tris buffer (100 mM, pH 7.2) for 30 min, and washed excessively until no enzyme leaching was observed. All the GOx-immobilized CNTs were suspended in 100 mM PB (pH 7.0) at the concentration of 0.5 mg/mL CNTs, and stored at 4 °C until use.

### Activity and stability measurements

The activity of immobilized GOx on CNTs was measured by a conventional GOx assay^63^. GOx-immobilized CNTs (10 μL, 0.5 mg/mL) were added to the mixture of 10% (w/v) D-glucose solution (980 μL) with 10 uL of 0.21 mM *o*-dianisidine and 6 units/mL horseradish peroxidase solution. GOx activity was determined by measuring the increase in absorbance at 500 nm over time by using a UV-Vis spectrophotometer (UV-1800, Shimadzu, Japan). One unit of GOx activity is defined by the amount of enzyme that catalyzes the oxidation of 1 μmole of glucose per min. GOx stability was determined by measuring residual enzyme activity after different times of incubation by using an aliquot from the stock solutions of free and immobilized enzymes under incubation at room temperature. The relative activity was calculated from the ratio of residual activity at each time point to the initial activity of each sample.

### Preparation of enzyme anodes, and operation of enzymatic biofuel cell

A CP electrode with a thickness of 0.37 mm and an area of 0.33 cm^2^ was used as the backing material for preparation of enzyme anodes. CP was treated with the mixed solution of sulfuric acid and hydrogen peroxide to enhance its hydrophilicity. The acid-treated CPs were immersed in 0.5% Nafion^®^ containing GOx immobilized by one of the two methods described above for 10 min, and dried under ambient conditions. The CP enzyme anodes were kept in 100 mM PB (pH 7.0) at least overnight before use.

An air-breathing home-made biofuel cell (2 × 2 cm) was used to test each enzyme anode, and the electrochemical measurements were performed using a Bio-Logic SP-150 potentiostat (BioLogic Science Instrument, Grenoble, France)^43^,^64^. The key components of a biofuel cell are the anode chamber, enzyme anode, current collector, and MEA. The MEA consists of a proton exchange membrane (Nafion^®^ 117) and a Pt cathode. Glucose solution (200 mM) in 100 mM PB (pH 7.0) was fed as a fuel to the anode chamber, while ambient air was provided to the cathode. Polarization curves were obtained using the constant load discharge (CLD) mode. In this manner an external load was applied to the cell from a resistance box while the current and voltage outputs were measured.

### Laviron simulation for electron transfer rate constants

The electron transfer rate constant of each enzyme electrode was estimated using Laviron’s model^55^. By adopting the model’s formula, two straight lines, (1) E_pa_ − E_0_ vs. log ν and (2) E_pc_ − E_0_ vs. log ν, were obtained. E_pa_ is the anodic peak potential, E_pc_ is the cathodic peak potential, log ν is the logarithm of the scan rate, and E_0_ is the average potential of anodic and cathodic peak potentials. The electron transfer rate constants could be calculated using the transfer coefficient (α), which is determined from the intercepts of the two straight line fits (the anodic and cathodic line fits)^65^.

## Additional Information

**How to cite this article**: Kim, B. C. *et al*. Fabrication of enzyme-based coatings on intact multi-walled carbon nanotubes as highly effective electrodes in biofuel cells. *Sci. Rep.* **7**, 40202; doi: 10.1038/srep40202 (2017).

**Publisher's note:** Springer Nature remains neutral with regard to jurisdictional claims in published maps and institutional affiliations.

## Supplementary Material

Supplementary Information

## Figures and Tables

**Table 1:** Comparison of CA and EAPC enzyme activities, electron transfer rate constants of enzyme-based electrodes, and maximum power densities of enzyme-containing anodes in biofuel cell operation.

	Activity (units/mg of CNTs)	Electron transfer rate constant (s^−1^)	Maximum power density (μW cm^−2^)
CA-GOx/ox-CNTs*	2.0	1.8	1.1
EAPC-GOx/CNTs**	9.0	3.1	7.9
Ratio (**/*)	**4.5**	**1.7**	**7.2**

The ratio of maximum power densities between the two formulations matches well with the combination of activity and electron transfer rate constant ratios.
