# Supplementary material for: Fabrication of enzyme-based coatings on intact multi-walled carbon nanotubes as highly effective electrodes in biofuel cells
Source: Sci Rep. 2017 Jan 5;7:40202. doi: 10.1038/srep40202 (PMC5215464; doi:10.1038/srep40202)
Supplement: Supplementary Information [file srep40202-s1.pdf]

Supporting Information

Fabrication of enzyme-based coatings on intact multi-walled carbon nanotubes as highly effective electrodes in biofuel cells

Byoung Chan Kim[1,2,+], Inseon Lee[3,+], Seok-Joon Kwon[4], Youngho Wee[3], Ki Young Kwon[5], Chulmin Jeon[3], Hyo Jin An[3], Hee-Tae Jung[5], Su Ha[6], Jonathan S. Dordick[4,*], Jungbae Kim[3,7,*]

[1] Center for Environment, Health and Welfare Research, Korea Institute of Science and Technology, Seoul 02792, Republic of Korea

[2] Department of Energy and Environmental Engineering, Korea University of Science and Technology (UST), Seoul 02792, Republic of Korea

[3] Department of Chemical and Biological Engineering, Korea University, Seoul 02841, Republic of Korea

[4] Department of Chemical and Biological Engineering, Rensselaer Polytechnic Institute, Troy, NY 12180, USA

[5] Department of Chemical and Biomolecular Engineering, Korea Advanced Institute of Science and Technology, Daejeon 34141, Republic of Korea

[6] Chemical Engineering Department, Washington State University, Pullman, WA 99164-2710, USA

[7] Green School, Korea University, Seoul 02841, Republic of Korea

[+] These authors contributed equally to this work.

*** Corresponding authors**

E-mail address: dordick@rpi.edu (J. S. Dordick), jbkim3@korea.ac.kr (J. Kim)

Supporting table and figures

	Thickness (nm)
CNTs	30 ± 10
ox-CNTs	35 ± 5
CA (CA-GOx/ox-CNTs)	36 ± 7
EAPC (EAPC-GOx/CNTs)	52 ± 13

Table S1. The thicknesses of CNTs, ox-CNTs, CA-GOx/ox-CNTs and EAPC-GOx/CNTs, which were estimated from their SEM images.

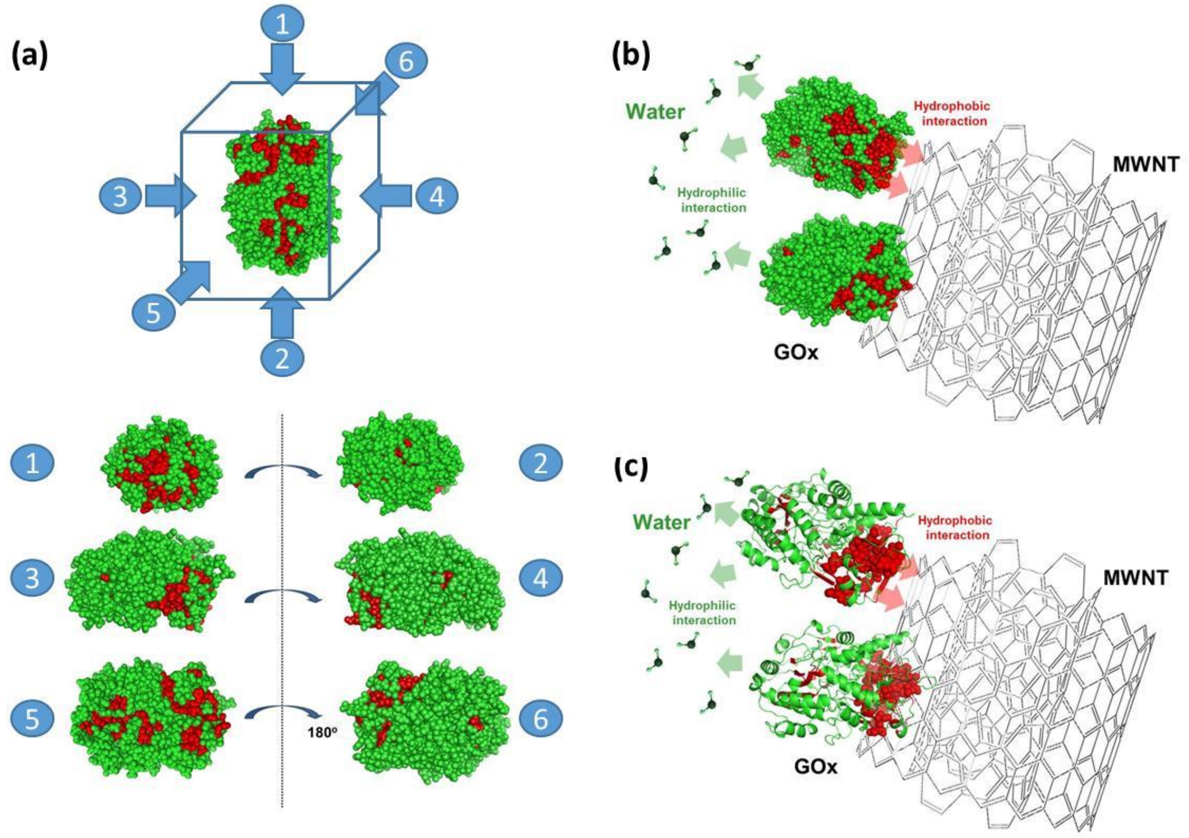

Figure S1. Illustration for hydrophobic interaction between CNT (MWNT) and GOx (PDB code: 1CF3) and hydrophilic interaction between GOx and water molecules. Hydrophobic side chains of valine, leucine, isoleucine and phenylalanine are indicated in red spheres, while the other parts in green. (a) Amphiphilic nature of GOx. A GOx molecule was viewed from six orthogonal directions, and the views from opposite directions (180°) are arranged side by side to show the amphiphilic nature of GOx. Especially, the top (1) and bottom (2) views vividly show the contrast of hydrophobic and hydrophilic surface characteristics by dominant red and green spheres, respectively. (b-c) The hydrophobic-dominant part (hydrophobic patch) on the surface of GOx from the top view in (a-1) would be able to interact with highly hydrophobic surface of intact CNT. Concomitantly, the hydrophilic side chains on the surface of GOx, represented by green spheres (b) and secondary structures (c), can interact with hydrophilic water molecules, which can help to disperse the intact CNTs in an aqueous GOx solution.

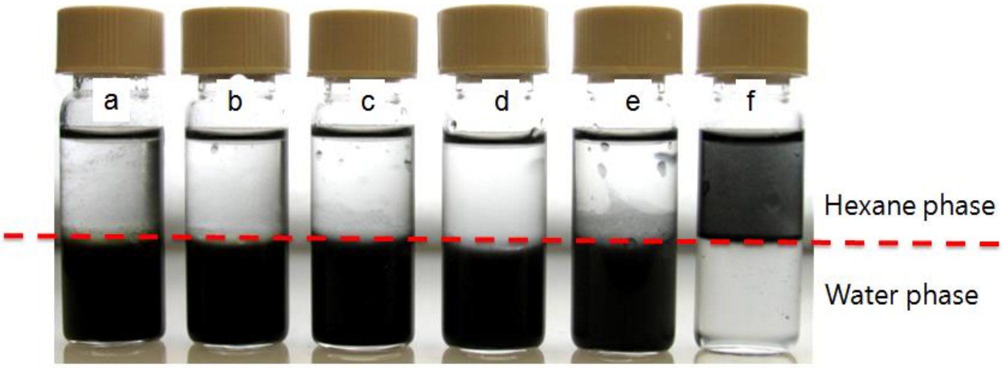

Figure S2. Dispersion and no extraction of CNTs in GOx solution. Intact CNTs (1.5 mg) were added to 100 mM PB (pH 7.0) containing GOx in various concentrations (10, 5, 1, 0.5, 0.1, and 0 mg/mL for a - f). After shaking at 200 rpm for 1 h, hexane was added, and the mixture was shaken at 200 rpm for 0.5 h. In the absence of GOx, all the intact CNTs were extracted into the hexane phase (f); however, even with the lowest GOx concentration (0.1 mg/mL), CNTs were not extracted to the hexane phase, and maintained their high dispersion in the aqueous phase.

(a)

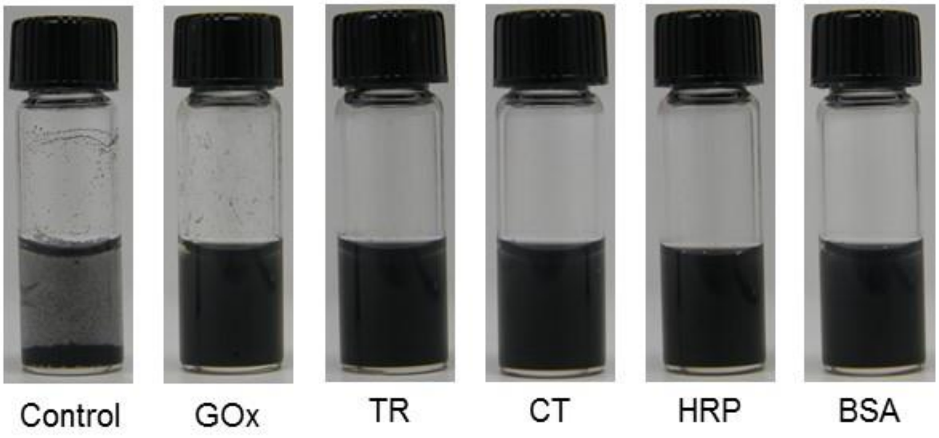

(b)

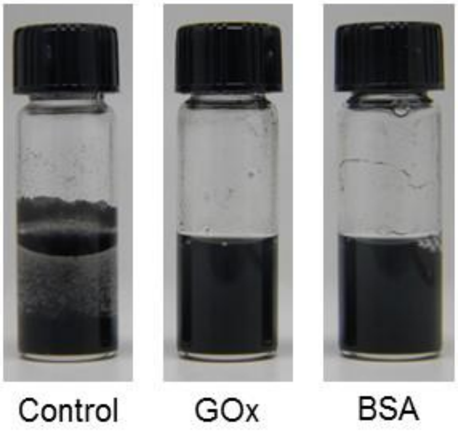

Figure S3. (a) Dispersion of CNTs in aqueous protein solutions. CNTs (multi-walled, 30 ± 15 nm in outer diameter and 1~5 μm in length, purity > 95%) were suspended in 1 mL of phosphate buffer (PB, 100 mM, pH 7.0), and this CNT suspension was mixed with 1 mL of protein solution (10 mg/mL protein in 100 mM PB, pH 7.0) under shaking (200 rpm) for 1 h. Then, the above pictures were taken after letting the bottles stand without shaking for 3 min. In the absence of protein (control), intact CNTs aggregated while CNTs were well dispersed in the presence of the proteins tested (glucose oxidase, GOx; trypsin, TR; chymotrypsin, CT; horseradish peroxidase, HRP; bovine serum albumin, BSA). **(b) Dispersion of graphene in aqueous protein solution.** Graphenes (3 nm in average flake thickness and 10 μm in average diameter) were selected to assess their dispersion in protein solutions consisting of GOx and BSA. Graphenes were suspended in 1 mL of phosphate buffer (PB, 100 mM, pH 7.0), and the graphene suspensions were mixed with 1 mL of protein solution (10 mg/mL protein in 100 mM PB,

pH 7.0) under shaking (200 rpm) for 1 h. Then, the above pictures were taken after letting the vials stand without shaking for 3 min. In the absence of protein (control), graphenes aggregated while they were well dispersed in the presence of GOx or BSA.

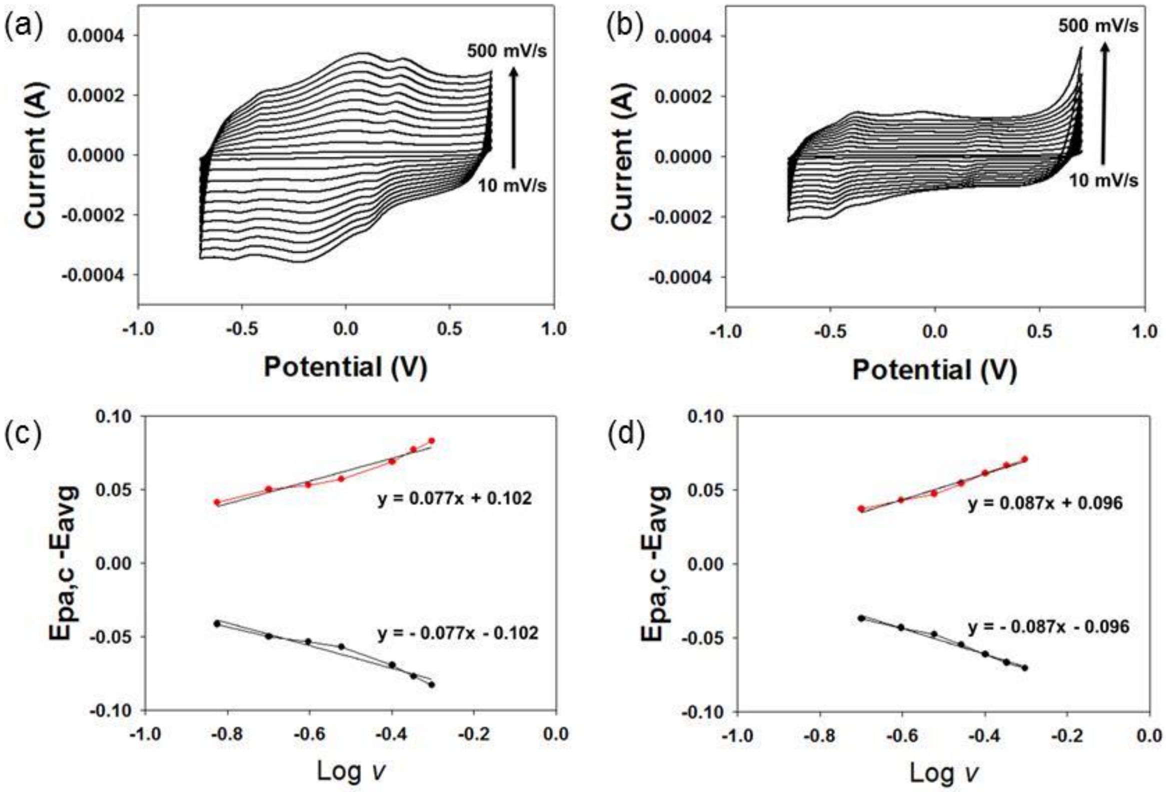

Figure S4. Cyclic voltammograms (CVs) and Laviron plots. CVs of CA-GOx/ox-CNTs (a) and EAPC-GOx/CNTs (b) obtained at different CV scan rates, ranging from 10 mV s^{-1} to 500 mV s^{-1}. Electron transfer rate constants were determined by Laviron plots of CA-GOx/ox-CNTs (c) and EAPC-GOx/CNTs (d).
